# An Unobtrusive Fall Detection and Alerting System Based on Kalman Filter and Bayes Network Classifier

**DOI:** 10.3390/s17061393

**Published:** 2017-06-16

**Authors:** Jian He, Shuang Bai, Xiaoyi Wang

**Affiliations:** 1 Beijing Advanced Innovation Center for Future Internet Technology, Beijing 100124, China; Jianhee@bjut.edu.cn; 2 Beijing Engineering Research Center for IoT Software and Systems, Beijing 100124, China; 3 School of Software Engineering, Beijing University of Technology, Beijing 100124, China; Baishuang@emails.bjut.edu.cn

**Keywords:** fall detection, Kalman filter, Bayes network classifier, smart phone, Bluetooth

## Abstract

Falls are one of the main health risks among the elderly. A fall detection system based on inertial sensors can automatically detect fall event and alert a caregiver for immediate assistance, so as to reduce injuries causing by falls. Nevertheless, most inertial sensor-based fall detection technologies have focused on the accuracy of detection while neglecting quantization noise caused by inertial sensor. In this paper, an activity model based on tri-axial acceleration and gyroscope is proposed, and the difference between activities of daily living (ADLs) and falls is analyzed. Meanwhile, a Kalman filter is proposed to preprocess the raw data so as to reduce noise. A sliding window and Bayes network classifier are introduced to develop a wearable fall detection system, which is composed of a wearable motion sensor and a smart phone. The experiment shows that the proposed system distinguishes simulated falls from ADLs with a high accuracy of 95.67%, while sensitivity and specificity are 99.0% and 95.0%, respectively. Furthermore, the smart phone can issue an alarm to caregivers so as to provide timely and accurate help for the elderly, as soon as the system detects a fall.

## 1. Introduction

Falls are one of the main health risks among the elderly due to the increase in mortality, morbidity, disability, and frailty [1]. Besides physical injuries, the fear of falling is developing among the elderly, which greatly reduces their confidence in living independently and participating energetically in social activities, ultimately resulting in significant reductions in the quality of their lives and contributing to an increase in frailty due to this reduction of activity levels [2]. Reports show that approximately 3% of all fallers lie for more than 20 min without external support, while 80% of the fallers aged 90 years or older are unable to get up by themselves [3]. Hence, an automatic notification to caregivers after detecting a fall will be greatly helpful for elderly by reducing the time of waiting for medical support after the fall.

Since falls are a major health risk among elderly, different kinds of methods have been developed to automatically detect falls in the last decade, which can be categorized into three different classes depending on the deployed sensor technology; namely, vision-based sensors, ambient sensors and wearable devices [4]. For example, Yu et al. developed a vision-based fall detection method by applying background subtraction to extract the foreground human body, and information is fed into a directed acyclic graph supporting vector machine (SVM) for posture recognition so as to detect falls [5]. Yazar et al. introduced vibration and passive infrared (PIR) sensors, and used Winner-takes-all decision algorithm to detect falls [6]. However, both vision-based and ambient sensors have a constrained monitoring area and conditions, which require installation and maintenance, leading to high costs. Recently, the advancements in microelectromechnical systems (MEMS) have brought about smaller and cheaper inertial sensors, which are widely used to develop wearable devices to measure physical activities under real-life environment, including very private areas like the bathroom [7]. Since smart phones integrated with inertial sensors are more and more popular, many works have investigated fall detection on the smartphone. For example, Bai et al. presented a system based on a tri-axial accelerometer embedded in a smart phone with global positioning system (GPS) function to detect falls [8]. Salgado introduced an extended Kalman filter (EKF) algorithm to identify the Pose Body Model (PBM), and used SVM to detect falls using smart phones [9]. However, the difference between smart phones in terms of the pre-set sample rate has an effect on its application for fall detection [10]. Besides, even though the inertial sensor is widely used in smart phones and wearable devices, it has non-negligible measurement noise. El-Sheimy et al. [11] presented analysis and modeling of inertial sensors using Allan variance. It shows that in the short cluster times, the quantization noise is the prominent error term, whereas this is the drift rate-ramp term in the long cluster times.

In this paper, a Kalman filter is introduced to develop an unobtrusive fall detection and alerting system, which can reduce the measurement noises from inertial sensors. The system consists of a smart phone, and a custom vest integrated with tri-axial accelerometer and gyroscope. The custom vest worn by an elderly person can sample the individual tri-axial accelerations and angular velocities, and send them to the smart phone via Bluetooth. The program running on the smart phone preprocesses the data by Kalman filter so as to reduce measurement noise, and then judges whether the individual is falling or not based on the Bayes network classifier. The smart phone can issue an alarm to caregivers so as to provide timely and accurate help for the elderly, as soon as it detects a fall.

The rest of this paper is organized as follows. Section 2 introduces the available technology for wearable fall detection. The methodology to model physical activities and extract the features is discussed in Section 3. Section 4 introduces the implementation of the system. The simulated experiment and its analysis are discussed in Section 5. The future research is presented in conclusion.

## 2. Related Work on Wearable Fall Detection 

In order to solve the problem of fall detection, different wearable devices based on inertial sensors have been explored, which vary in sensor type, placement, device, quantity and approach. The majority of these devices can be divided into three main types according to the main approaches: threshold-based, threshold combining with phase, and machine learning [12]. 

Threshold-based fall detection approaches use single or multiple thresholds to extract features. Bourke et al. [13] introduced an approach for detecting falls based on the assumption that acceleration in falls is sharper than those in ADLs. Lindemann et al. [14] integrated a hearing aid device with a tri-axial accelerometer, and detected falls by thresholds for acceleration and velocity. Wang et al. [15] applied a tri-axial accelerometer and wireless sensor network to develop an enhanced fall detection system for monitoring of the elderly. The main problem was that the system, using only acceleration, led to many false positives. For instance, the vertical acceleration data produced by sitting down quickly are similar with those in falls. Hence, more and more researchers study technology on combining tri-axial accelerometers with gyroscopes so as to detect fall events accurately.

Researchers have also combined threshold with phase to detect falls. Li et al. [16] proposed a system with a three phase model to detect falls. Two accelerometers are placed on the abdomen and the right thigh, and the data stream is segmented into one-second windows in the system. The first phase monitors whether the user is static or dynamic, the second phase recognizes the lying state and the last phase is to judge whether the transition is intentional or not. They collect typical acceleration amplitude and rotational rate, and find that it is less than 0.4 g and 60° respectively. The lying state is identified when the angle between the gravitational vector and the trunk, and the angle between the gravitational vector and the thigh are larger than the threshold. The final phase, checking for intention, is determined by identifying when the peak value within a window is larger than the threshold. The algorithm could reduce false alarms by deriving the posture information from both gyroscopes and accelerometers. Gjoreski et al. [17] proposed RAReFall (Real-Time Activity Recognition and Fall Detection System) which measures the difference between the maximum value and minimum value within a one-second window; if the difference is larger than 1 g and the maximum value occurs after the minimum value, then a fall is detected.

Machine learning approaches use automatic technology which starts from the extracted features, and tries to distinguish falls from ADLs [18]. Ojetola et al. [19] introduce two sensor motes (each has one accelerometer and one gyroscope) worn on the right thigh and chest to distinguish falls from ADLs. In this system, raw data is first processed by mean filter and lower resampling, then using features such as vector magnitude of acceleration and angular velocity to train a C4.5 decision tree model. Zhang et al. [20] proposed a fall detection method based on a one class SVM which uses a tri-axial accelerometer to capture human movement data. Since it needs specific activity patterns and computation, it is not appropriate for detecting falls in real time. Tong et al. [21] used a hidden Markov model (HMM) and tri-axial accelerometer to detect and predict falls, and the experimental results showed that, using the system, falls could be predicted 200–400 ms before impact, and could also be accurately distinguished from other daily activities. However, the HMM λ (which is introduced to describe fall process) and thresholds of the system were set according to data samples of young people’s simulated activities; the mathematical model and thresholds should be trained and reset according to the large real-world samples of the elderly. Dinh and Struck [22] transformed acceleration data from Cartesian coordinates to spherical coordinates, and developed an algorithm based on a neural network and fuzzy logic to detect falls.

Overall, one of main challenges for wearable fall detection is the lack of agreement among research groups. For example, different sampling frequencies were used for sampling from the accelerometers, with some using rather low frequencies of less than 20 Hz. Sensor placement positions vary, and include waist, wrist, hip, and trunk attachment [23]. Besides, though inertial sensors are very suitable for wearable devices because of their small size and low cost, they have non-negligible measurement noise, which could affect the measuring accuracy of the monitoring object. Ligorio and Sabatini used a linear Kalman filter to fuse the tri-axial gyroscope and accelerometer data. The analysis of the experiment showed that significant accuracy improvement was achieved over state-of-the-art approaches, because a filter design better matched the basic optimality assumptions of Kalman filtering [24]. As a result, a Kalman filter is introduced to preprocess the raw tri-axial gyroscope and accelerometer data so as to reduce the measurement noise caused by inertial sensors. 

## 3. Methodology

Research shows that the upper trunk is the most appropriate feature region for identifying falls from other movements by acceleration [25]. Meanwhile, in order to reduce the inconvenience caused by wearable devices, and protect the sensor board from being broken, the sensor board is put on the top of the customer’s vest.

### 3.1. Model Activity

**Figure 1:**
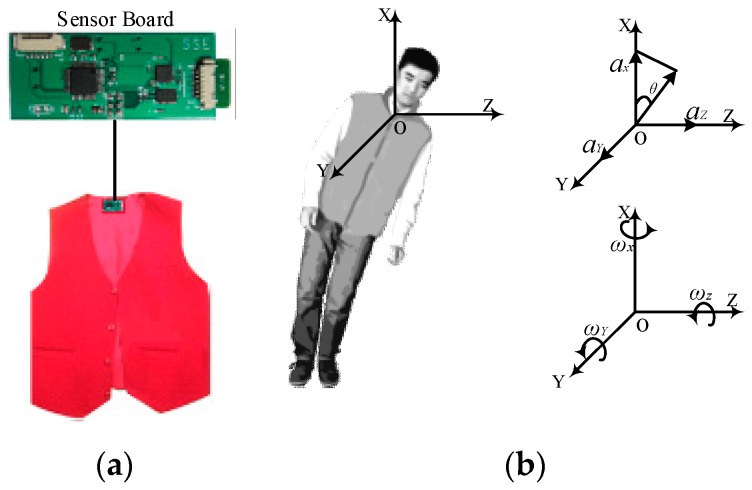
(**a**) The placement of the sensor board; (**b**) The geodetic coordinate OXYZ.

Based on the fact that the direction of gravity is invariably perpendicular to the ground, and that the orientation of the vest worn on the body is supposed to be the same as that of the trunk, we use a Cartesian coordinate system OXYZ for the upper trunk, the origin of which is close to the neck of the human body, and is parallel with the geodetic coordinate system OXYZ, as shown in Figure 1. 

At a time *t*, accelerations along the *X*, *Y*, and *Z* axes are denoted as *α_x_(t)*, *α_y_(t)* and *α_z_(t)* respectively, namely *α(t)* = {*α_x_(t), α_y_(t), α_z_(t)*}. The resultant acceleration *α(t)* can be calculated using Equation (1):

(1) $$a(t)=\sqrt{a_{x}{\left(t\right)}^{2}+a_{y}{\left(t\right)}^{2}+a_{z}{\left(t\right)}^{2}}$$

Since α_x_*(t)*, α_y_*(t)* and α_z_*(t)* contain an approximation of the gravitational component of the acceleration on every axis, the trunk angle (namely *θ(t)*) can be calculated using Equation (2):

(2) $$\theta (t)=\cos^{-1}\left(\frac{a_{x}(t)}{\sqrt{\alpha_{x}{\left(t\right)}^{2}+\alpha_{y}{\left(t\right)}^{2}+\alpha_{z}{\left(t\right)}^{2}}}\right)$$

The *x*-axis is perpendicular to the gravitational direction in the lying position and parallel to the gravitational direction in the standing position. A fall usually means that the trunk changes from a standing position to a lying position, and the *θ(t)* increases from about 0° to about 90°.

Meanwhile, the tri-axial angular velocities of the trunk can be collected by gyroscope. *ω_x_(t)*, *ω_y_(t)*, and *ω_z_(t)* are the angular velocity at time *t* in the *X*, *Y*, and *Z* axes respectively, namely *ω(t)* = {*ω_x_(t)*, *ω_y_(t), ω_z_(t)*}. The resultant angular velocity *ω(t)* can be calculated using Equation (3):

(3) $$\omega (t)=\sqrt{\omega_{x}{\left(t\right)}^{2}+\omega_{y}{\left(t\right)}^{2}+\omega_{z}{\left(t\right)}^{2}}$$

### 3.2. Data Acquisition

**Figure 2:**
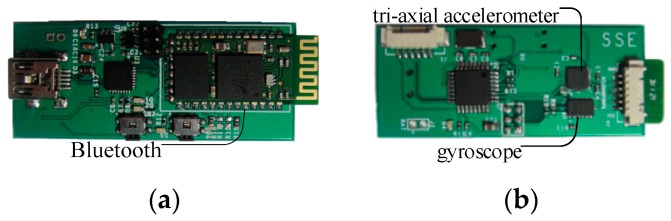
(**a**) Front of the sensor board with Bluetooth; (**b**) Back of the sensor board with tri-axial accelerometer and gyroscope.

Figure 2 shows the sensor board, which is about 65 mm × 40 mm × 7 mm (length × width × thickness), and is appropriate for use in a vest. The sensor board contains a low-power microcontroller, and a class 2 Bluetooth module. The range of the Bluetooth module is 10 m, and its default transmission rate is 115,200 bps. The range of the tri-axial accelerometer is ±16 g. The full-scale range of the tri-axial gyroscope is ±2000°/s. The sampling data from the tri-axial accelerometer and gyroscope are read and transmitted to an Android smart phone.

Since most frequencies of human activities are less than 20 Hz, the sampling frequency from human activities is set to 100 Hz. The sensor board can acquire tri-axial accelerations and angular velocities, and send them directly to an Android smart phone.

Since falls are usually characterized by rapid acceleration and great angular velocity, four typical subcategories of ADLs and two kinds of falls are proposed in order to find out the difference between ADLs and falls. ADLs include Walking (Wk), Sitting down (Sd), Squatting down (Sq) and Bowing (Bw). Falls include Sideward fall (Sw-Fall) and Backward fall (Bw-Fall). Twenty healthy individuals, including ten males and ten females, aged 20–45 years were asked to do the simulated falls and normal ADLs, both outdoors and indoors. Each individual performs each of the six kinds of ADLs and falls—Wk, Sd, Sq, Bw, Sw-Fall and Bw-Fall—five times. Hence, the total experimental data set numbers 600 elements, which consists of six 100-element sets for Bw-Fall, Sd-Fall, Wk, Sd, Sq and Bw, respectively, with each one having the same sample length.

### 3.3. Filter Noise

Being a linear quadratic estimation (LQE), the Kalman filter uses a series of measurements observed over time, containing statistical noise and other inaccuracies, and produces estimates of unknown variables that tend to be more precise than those based on a single measurement alone. The Kalman filter is widely applied in time series analysis, such as signal processing and trajectory optimization [26]. Because ADLs and falls are discrete time-finite dimensional linear stochastic processes, and MEMS-based inertial sensors have non-negligible measurement noise, a Kalman filter is introduced to reduce the noise so as to improve the accuracy of monitoring state.

The software running on the Android smart phone receives the angular velocities and tri-axial accelerations from the sensor board, then it preprocesses the data through the Kalman filter. The main process for preprocessing the tri-axial accelerations and angular velocities is as follows.

Predict the state value at time *k* using Equation (4):

*X(k|k* − *1) = AX(k* − *1|k* − *1) + BU(k)* (4)

Calculate the prediction error covariance matrix at time *k* using Equation (5):

*P(k|k* − *1) = AP(k* − *1|k* − *1)A^T^ + Q* (5)

Kalman gain at time *k* (namely *Kg*(*k*)) is calculated using Equation (6):

(6) $$Kg\left(k\right)=\frac{P(k|k-1)H^{T}}{HP(k|k-1)H^{T}+R}$$

By combining the predicted values *X(k|k* − *1)* with the measured values *Z(k)*, the optimal estimate *X(k|k)* at time *k* can be obtained using Equation (7):

*X(k|k) = X(k|k* − *1) + Kg(k)[Z(k)* − *HX(k|k* − *1)]* (7)

Update the covariance matrix of the estimation error at time *K* using Equation (8):

*P(k|k) = (I* − *Kg(k)H)P(k|k* − *1)* (8)

where, *I* is an identity matrix; *A* is the state transition matrix of the process of human movement from the state at *k* − *1* to the state at *k*; *B* is the control input model; *U* is a deterministic process input, which, Since ADLs and falls are linear stochastic processes, the input vector *U* will be a zero vector; *A^T^* refers to the transpose of the matrix; *H* is the noiseless connection between the state vector and the measurement vector, which is an identity matrix because the state variables are observed directly; *R* is the covariance matrix of the observation noise; and *Q* is the covariance matrix of the measurement noise. The sensor board has been statically placed on the ground for 2 min, and the tri-axial accelerations and angular velocities have been sampled from the sensor board to build the autoregressive (AR) model [27], so as to calculate *A*, and the initial values for both *Q* and *R*.

Table 1 shows the parameters and final prediction error (FPE) of the tri-axial accelerations for the one order AR model (AR(1)), two order AR model (AR(2)) and three order AR model (AR(3)). It can be seen from Table 1 that the difference of FPE among AR(1), AR(2) and AR(3) is very tiny in terms of tri-axial acceleration. Hence, AR(1) is selected to describe the time-varying processes for tri-axial accelerations.

According to the AR(1) and FPE of the tri-axial acceleration, the *A* and the initial value both for *Q* and *R* can be obtained as follows.

$$A=\lceil \begin{array}{ccc} 0.9974 & 0 & 0\\ 0 & 1 & 0\\ 0 & 0 & 0.9953 \end{array}\rceil ,\;Q=\lceil \begin{array}{ccc} 4.3\times 10^{-5} & 0 & 0\\ 0 & 3.29\times 10^{-5} & 0\\ 0 & 0 & 5.38\times 10^{-5} \end{array}\rceil ,\;R=\lceil \begin{array}{ccc} 0.00002 & 0 & 0\\ 0 & 0.00002 & 0\\ 0 & 0 & 0.00003 \end{array}\rceil$$

Table 2 shows the AR parameters and FPE for tri-axial angular velocities. Table 2 shows that the difference of FPE among AR(1), AR(2) and AR(3) is very tiny in tri-axial angular velocities. Therefore, AR(1) was also selected to model the time-varying processes for tri-axial angular velocities as well.

According to the AR(1) and FPE of the tri-axial angular velocities, the *A* and initial value for both *Q* and *R* can be calculated as follows.

$$A=\lceil \begin{array}{ccc} 1 & 0 & 0\\ 0 & 0.9269 & 0\\ 0 & 0 & 0.9997 \end{array}\rceil ,\;Q=\lceil \begin{array}{ccc} 0.0012 & 0 & 0\\ 0 & 0.0010 & 0\\ 0 & 0 & 0.0011 \end{array}\rceil ,\;R=\lceil \begin{array}{ccc} 0.002 & 0 & 0\\ 0 & 0.00087 & 0\\ 0 & 0 & 0.00109 \end{array}\rceil$$

**Figure 3:**
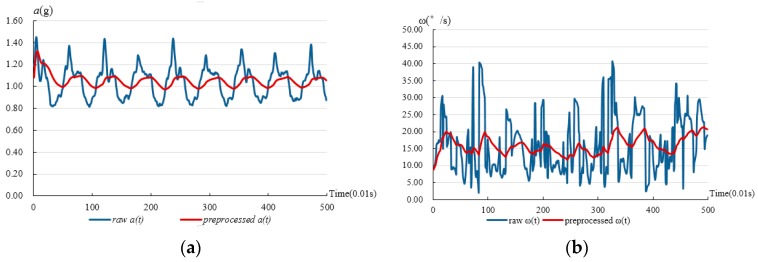
(**a**) Comparison between raw and preprocessed resultant accelerations from normal walking; (**b**) Comparison between raw and preprocessed resultant angular velocities from normal walking.

Figure 3 shows the comparison between the raw and preprocessed data of the resultant acceleration and angular velocity from a normal walk. It can be observed from the curve that the Kalman filter can eliminate jitters of the curve both in the resultant acceleration and angular velocity, and it will helpfully extract the features of the fall and ADLs. Additionally, Figure 3 also shows that the resultant acceleration is always above 1 G (where G is the gravity acceleration constant).

### 3.4. ADLs vs. Falls

**Figure 4:**
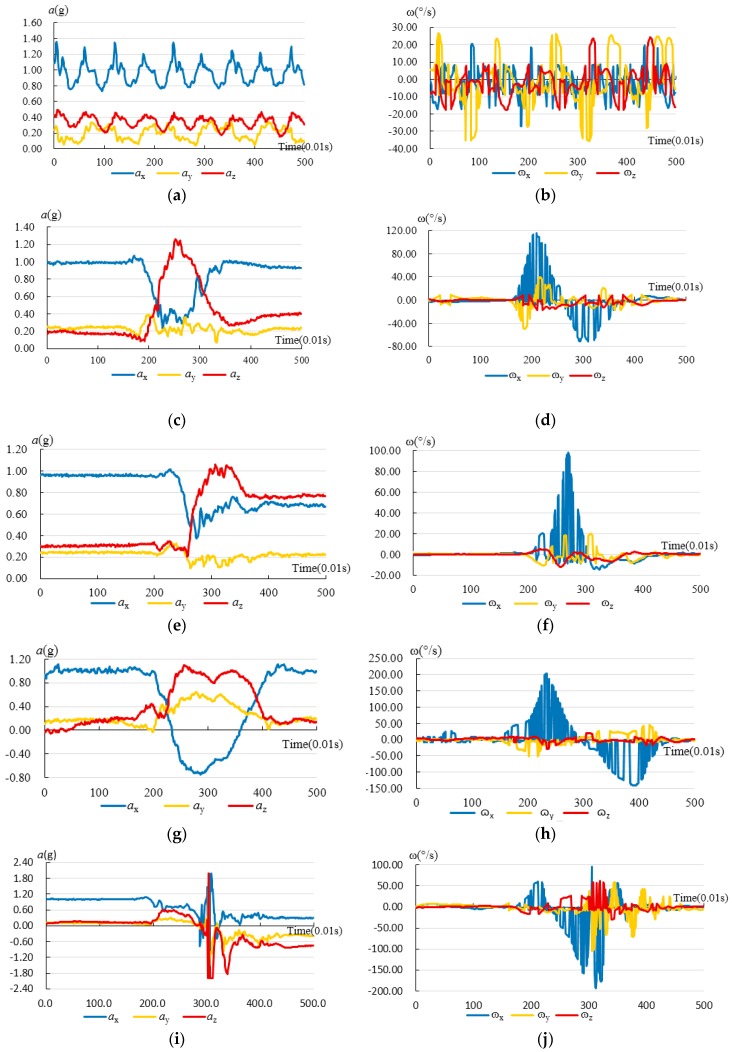

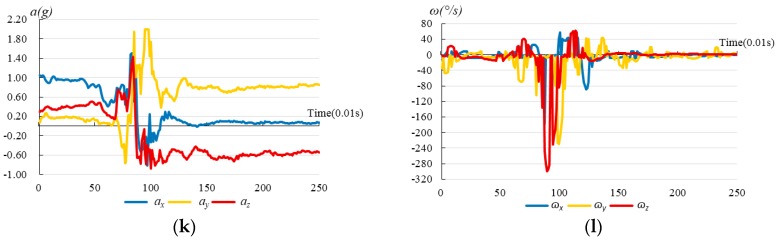
Comparisons of curves for tri-axial acceleration and angular velocity with Kalman filter from ADLs and falls: (**a**) Curve of the tri-axial accelerations from Wk; (**b**) Curve of the tri-axial angular velocities from Wk; (**c**) Curve of the tri-axial accelerations from Sd; (**d**) Curve of the tri-axial angular velocities from Sd; (**e**) Curve of the tri-axial accelerations from Sq; (**f**) Curve of the tri-axial angular velocities from Sq; (**g**) Curve of the tri-axial accelerations from Bw; (**h**) Curve of the tri-axial angular velocities from Bw; (**i**) Curve of the tri-axial accelerations from Bw-Fall; (**j**) Curve of the tri-axial angular velocities from Bw-Fall; (**k**) Curve of the tri-axial accelerations from Sd-Fall; (**l**) Curve of the tri-axial angular velocities from Sd-Fall.

Figure 4 shows curves of preprocessed tri-axial accelerations and angular velocities from ADLs and Bw-Fall. The data preprocessed by Kalman filter comes from a young and male subject who is about 25 years old. The mass and height of the subject are 65 kg and 174 cm, respectively. So the data shown in Figure 4 are average data. The horizontal axis is for time, with a unit of 0.01 s, while the longitudinal axis is for tri-axial accelerations (namely *α_x_(t), α_y_(t)* and *α_z_(t)*) in the left part of the figure, and angular velocities (namely *ω_x_(t), ω_y_(t), and ω_z_(t)*) in the right part of the figure.

Figure 4 indicates significant distinctions among each kind of motion process. For instance, Figure 4a,b indicate that both the tri-axial accelerations and angular velocities of normal walking have some periodic features. In terms of acceleration, the *α_x_(t)* and *α*_z_*(t)* change sharply for Sd and Sq. The *α_x_(t), α_y_(t)* and *α*_z_*(t)* change greatly for Bw. However, the *α_x_(t), α_y_(t)* and *α*_z_*(t)* change sharply and quickly for Bw-Fall. In particular, the *α_z_(t)* changes most sharply for Bw-Fall. The peak values of the tri-axial accelerations are almost 2 G for Bw-Fall. In terms of angular velocity, the *ω*_x_*(t)* changes sharply for Sd, Sq, and Bw*,* while *ω*_z_*(t)* changes trivially. However, *ω*_x_*(t)*, *ω*_y_*(t)*, and *ω*_z_*(t)* change sharply for Bw-Fall. In particular, the *ω*_x_*(t)* changes most sharply for Bw-Fall. Since falls are usually characterized by rapid accelerations and great angular velocities, they could be distinguished from ADLs, as long as the relevant features are extracted.

### 3.5. Feature Extraction

In the process of human activities, tri-axial accelerations and angular velocities change in real-time, forming stream data. It is a great challenge to classify stream data with infinite length. A sliding window, taking only the last-seen *N* elements of the stream into account, is introduced so as to overcome the problem.

**Figure 5:**
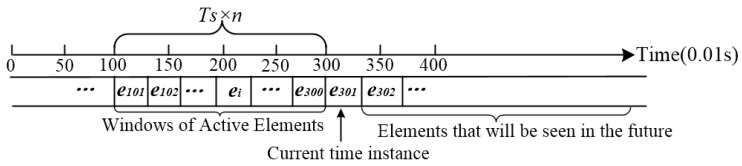
Illustration of the principles behind the sliding window.

Figure 5 illustrates the conventions of sliding windows; that the elements to the left are the ones that have already been seen, and that new data elements come from the right. The sliding window covers a time period of *T_S_ × n*, where *T_S_* is the same sampling period, and *n* is the number of sampling period. Each element in the sensor data stream has an arrival time, which increments by one at each arrival. The leftmost element is considered to have arrived at time 0. Since the duration of a fall is less than 2 s, *n* is set to 2. Meanwhile, the sampling frequency is set to 100 Hz. As a result, the sliding window has a length of 200.

For an explanation of this notation, consider the situation presented in Figure 5. The index of start time in the sliding window is 101, the index of current time instant is 301, and the last-seen element of the stream data is *e_300_*. Each element *e_i_* is a vector which consists of features used to classify falls. For example, in J. He’s paper [3], the resultant acceleration *a*, and angular velocity *ω* are selected as features to classify falls. Hence, *e_i_* = {*a_i_*, *ω_i_*}.

Algorithm 1 represents the program code for how the sliding window slides through the data stream, and the Bayes network distinguishes falls from ADLs. D_train_ is the training dataset for fall detection. Bayes Network is the classifier which compares the elements in the sliding window with D_train_, so as to identify falls from ADLs.

**Algorithm 1** Pseudo-Code Based on the Sliding Window and Bayes Network1**Input**: Sensor data stream2**Output**: Type(label) of a slide instance3label=4S_width_=200, //set the width of sliding window5**for** (Sref = 0; size (Sref+Swidth) ≥ Swidth; t ++)6label= Bayes network (D_train_, Sref + Swidth)7**end for**8return label

## 4. Implementation

Lots of smart devices (such as tablets and smart phones) are also integrated with Bluetooth modules as well, and have strong computing capabilities. Hence, a smart phone integrated with Bluetooth is used to receive the stream data from the sensors, and a program based on the above technologies and Bayes network classifier is developed to detect fall and issue alarm.

### 4.1. Bayes Network Classifier

Being a simple probabilistic classifier based on utilization of Bayes’ theorem with a strong independence assumption [28], the Bayes network classifier only requires a small amount of training data to estimate the parameters necessary for classification. It has worked pretty well in knowledge representation and inference engine both in artificial and expert systems [29].

A Bayesian network is a directed acyclic graph (DAG) that represents a joint probability distribution over a set of random variables *U.* Formally, *U* in a Bayesian network is defined by a pair *B* = <*G*,Θ>. The first component, namely *G*, is a DAG whose vertices correspond to the random variables *X_1_*, *X_2_*, …, *X_n_*, and whose edges represent direct dependencies between the variables. The graph *G* encodes independence assumptions, by which each variable *X_i_* is independent of its nondescendants given its parents in *G*. The second component, namely Θ, represents the set of parameters which quantifies the network. It includes a parameter $\theta_{x_{i}|\pi_{x_{i}}}$ = *P_B_*(*x_i_*|$\pi_{x_{i}}$) for each possible value *x_i_* of *X_i_*, and $\pi_{x_{i}}$ of $\pi_{x_{i}}$, where $\pi_{x_{i}}$ denotes the set of parents of *X_i_* in *G*. If *X_i_* has no parents, its local probability distribution is said to be unconditional, otherwise it is conditional. If the variable represented by a node is observed, then the node is said to be an evidence node, otherwise the node is said to be hidden or latent. Accordingly, a Bayesian network *B* defines a unique joint probability distribution (JPB) over *U*, namely:

(9) $$PB\left(X1,X2,\ldots ,Xn\right)=\prod_{i=1}^{n}P_{B}(X_{i}|\pi_{i})=\prod_{i=1}^{n}\theta_{X_{i}|\pi_{i}}$$

The problem of a Bayesian network classifier can be informally stated as: Given a training set *D* = (***u****_1_*, …, ***u****_N_*) of instances of *U*, find a network *B* that best matches *D*. The common approach to this problem is to introduce a scoring function that evaluates each network with respect to the training data, and then to search for the best network according to this function.

### 4.2. Software Design

The software consists of an on-chip program running on the sensor board, and the fall detection program running on an Android smart phone. The key steps of the on-chip program are as follows.

- Initialize the gyroscope and tri-axial accelerator, set the sampling frequency for the angular velocities, tri-axial accelerations and the baud rate for Bluetooth.
- Sample the angular velocities and accelerations from gyroscope and tri-axial accelerometer at an interval of 0.01 s.
- Send the angular velocities and tri-axial accelerations to the Android smartphone via Bluetooth.

**Figure 6:**
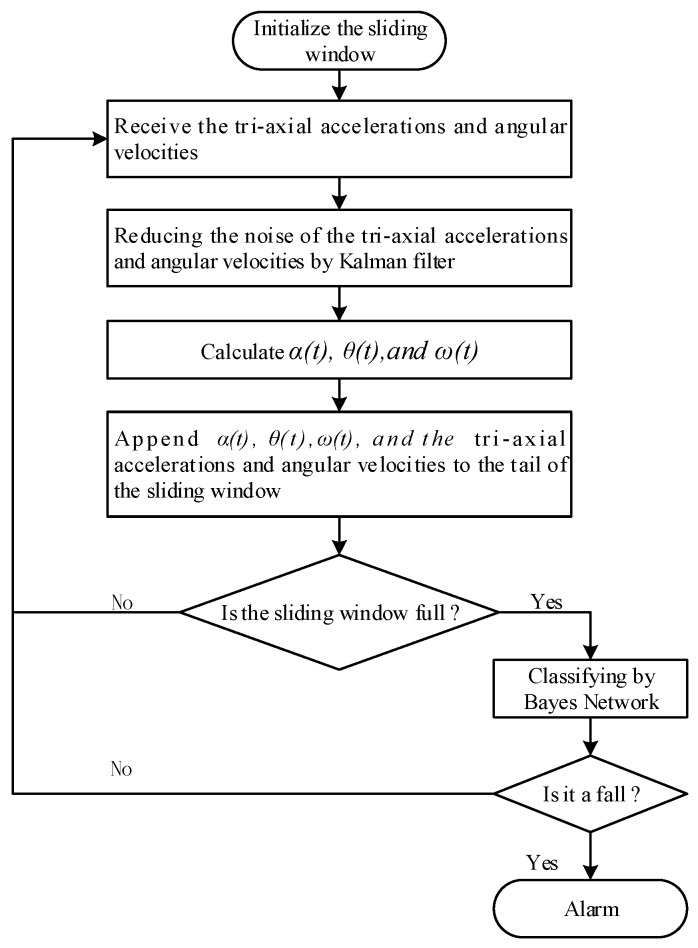
The flow chart of fall detection based on a naïve Bayes classifier.

Figure 6 indicates the flow chart of the program for fall detection that runs on an Android smart phone. The Android smart phone receives tri-axial accelerations (namely *a_x_(t)*, *a_y_(t)*, *a_z_(t)*) and angular velocities (i.e., *ω_x_(t)*, *ω_y_(t)*, *ω_z_(t)*) from the sensor board via Bluetooth. The resultant acceleration *α(t)*, trunk angle *θ(t)* and resultant angular velocity *ω(t)* are calculated according to Equations (1)–(3) respectively. The tri-axial accelerations and angular velocities, *α(t)*, *θ(t)* and *ω(t)* are appended to the tail of the sliding window. Based on the instance of a sliding window from the input stream, the program classifies the instance by Bayes network classifier. If the instance is classified as a fall pattern, the program will send an alarm. Otherwise, it is not classified as a fall pattern.

### 4.3. Software Implementation

**Figure 7:**
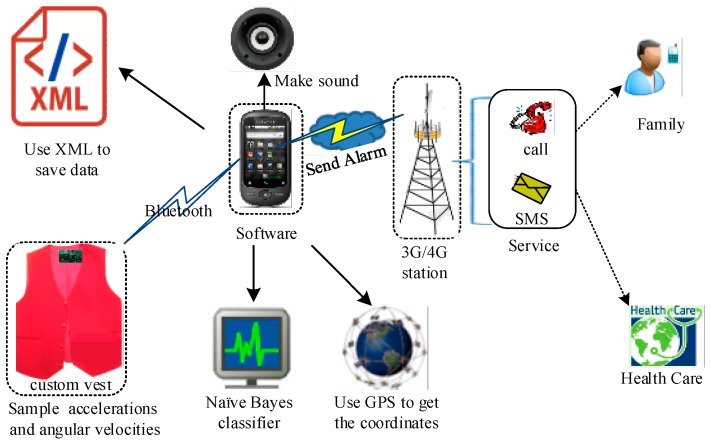
The fall detection system.

**Figure 8:**
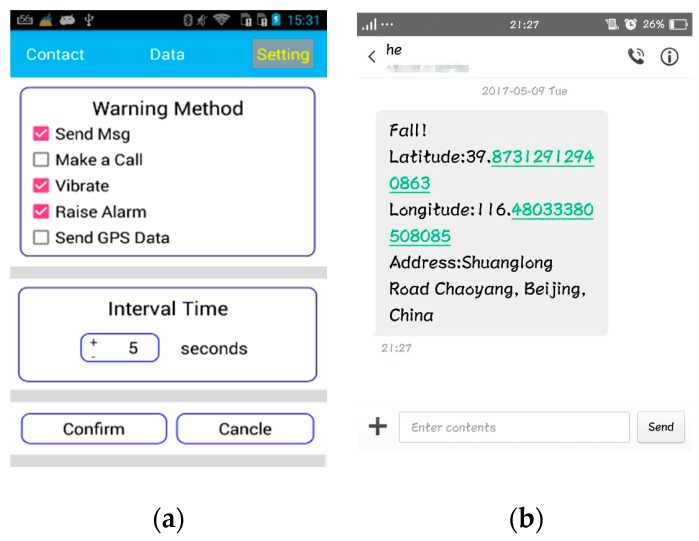
(**a**) Option menu to configure; (**b**) An alarm message with GPS location.

Figure 7 indicates the architecture of the fall detection system. The software which runs on the Android smart phone consists of several components: an XML (Extensive Markup Language) file to store the information received from custom vest, a Bayes network classifier that monitors the angular velocities and accelerations and judges whether a fall has occurred or not, and GPS to get the coordinates. In the event of a fall, the software will connect to a 3 G/4 G service that has a protocol for sending SMS messages or calls to a caregiver (namely family member or healthcare provider). In Figure 8a shows the interface that the user can use both to set the interval time for sending automatic notifications to caregivers after detecting a fall, and to configure different warning methods on the smart phone.

Each detected fall triggers an alarm. If the user cannot stop the alarm during the interval time, a call to a caregiver is made, or an emergency message including GPS location is immediately sent to caregivers, so as to provide timely and accurate help. Figure 8b shows an instance of an alerting message including GPS location when He fell down, and could not stop the alarm within the interval time.

## 5. Experiment

It is very dangerous for the elderly to test falls, so there is no experiment on elderly people over 50 years old. Twenty healthy individuals (i.e., 10 males and 10 females) aged from 20 to 45 years were asked to do the normal ADLs and simulated falls both outdoors and indoors. The average mass and height of volunteers were 64.5 kg and 172.3 cm, respectively. In accordance with the fall simulation protocol [30], fall simulation was conducted onto a spongy cushion of 15 cm thickness (hardness equal to 4 kPa pressure required to compress a piece of foam by 35% of its original height) to reduce the impact. Participants stood at a distance 1.5 times the length of their foot away from the spongy cushion, and were instructed to do Sd-Fall (or Bw-Fall) like a frail elderly person. There was no warm up trial to familiarize participants with the spongy cushion.

### 5.1. Experiment Results

There is a 100-element set for Bw-Fall, Sd-Fall, Wk, Sd, Sq and Bw, respectively, resulting in a total 600-element set of experimental data. The 10-fold cross-validation was introduced to the experiment and the algorithm had to classify six types of actions, rather than just judge whether a fall occurs or not. The experimental results with Kalman filter are indicated in Table 3. Table 4 shows the experimental results with raw data. In both Table 3 and Table 4, there are 9 features at each time point, namely *e_i_* = {*a_x_(t), a_y_(t), a_z_(t), a(t), ω_x_(t), ω_y_(t), ω_z_(t), ω(t), θ(t)*}. Table 3 shows that most samples were detected successfully, with only a small number of samples going undetected. It can be calculated from Table 5 that the accuracy was 95.67%, while the sensitivity and specificity were 99%, 95% respectively. On the contrary, it can be calculated from Table 4 that the accuracy was 94%, while sensitivity and specificity were 98% and 93.2% respectively. This proves that the algorithm coupled with Kalman filter reduces both false negatives and false positives, while improving the accuracy of fall detection.

Compared with traditional threshold-based methods using accelerations or gyroscopes at several single time points, the technology proposed in this paper is more effective for human fall detection. Most threshold-based methods use the results of sensing information at non-continuous time points to detect falls, thus some misdetection may be caused by the incompleteness of theinformation in experiments. For example, Li introduced a dynamic time-warping algorithm to develop fall detection system. This system, which didn’t distinguish Bw-Fall from Sd-Fall, achieved a sensitivity of 91% and a specificity of 92% [16]. In this paper, the new method reduces the noise for raw tri-axial acceleration and angular velocity using a Kalman filter, and analyzes the stream data throughout the whole course of the human fall process in a 2 s sliding window, so more sensing features are selected to identify falls from ADLs. As a result, the experiment shows better results.

Additionally, the Bayes network classifier with different features of activity at each time point are compared in terms of their accuracy, sensitivity, true positive (TP), false positive (FP), and running time. Table 5 shows a comparison of the experimental results with 9 features, namely *e_i_* = {*a_x_(t), a_y_(t), a_z_(t), a(t), ω_x_(t), ω_y_(t), ω_z_(t),ω(t), θ(t)*}; 7 features, namely *e_i_* = {*a_x_(t), a_y_(t), a_z_(t), ω_x_(t), ω_y_(t), ω_z_(t), θ(t)*}; and 3 features, namely *e_i_* = {*a(t),ω(t), θ(t)*}. It can be seen from Table 5 that a Bayes network classifier with 9 features has the highest accuracy, while the one with 3 features has the lowest accuracy. In other words, the more features are selected, the higher the accuracy the Bayes network classifier can achieve.

Finally, Weka (waikato environment for knowledge analysis) integration with various machine-learning algorithms was introduced to compare the Bayes network with other learning algorithms based on the same training dataset and test data. A Lenovo ThinkCenter m6200t with an i5 CPU and 4G memory was selected to run Weka. The comparison shown in Table 6 shows that both *k*-NN and the naïve Bayes algorithm take less than 0.3 s with accuracy of 95.5%. Additionally, the naïve Bayes algorithm has the highest sensitivity (namely 99.50%), but its accuracy and specificity are lower than *k*-NN and the Bayes network. The Bayes network has the highest accuracy, at 96.67%, and it takes about 1.33 s to classify the data. The C4.5 decision tree and Bagging have accuracies of 92.33% and 92.17%, respectively. They take more time to classify the falls, especially C4.5 which takes more than 6 s.

### 5.2. Discussion

**Figure 9:**
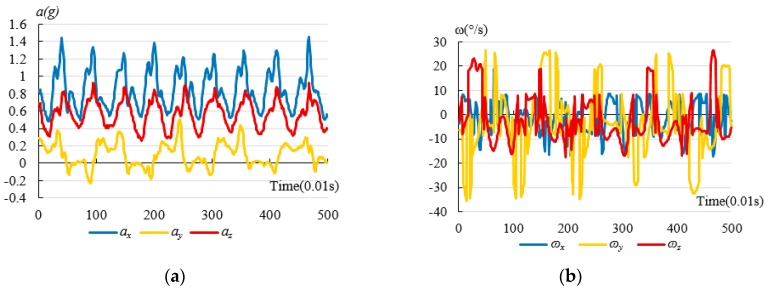

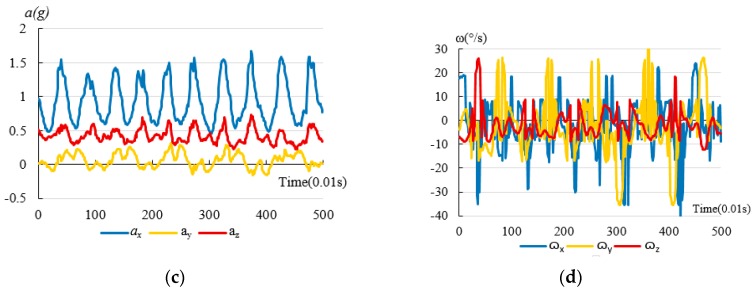
The curves of tri-axial accelerations and angular velocities with Kalman filter in Stair up and Stair down: (**a**) Curve of the tri-axial accelerations in Stair up; (**b**) Curve of the tri-axial angular velocities in Stair up; (**c**) Curve of the tri-axial accelerations in Stair down; (**d**) Curve of the tri-axial angular velocities in Stair down.

During design, the typical subcategories of ADLs, the physical conditions of the elderly, and their ordinary activities were carefully considered. For example, since jogging and jumping are unsuitable for elderly, they were not included in the subcategories of ADLs. Meanwhile, Stairs up and Stairs down were also compared with Wk. Figure 9 shows the curve of tri-axial accelerations and angular velocities from Stair up and Stair down, for which the subject is the same as in Figure 5. Figure 9 indicates that both the tri-axial accelerations and angular velocities for Stair up and Stair down have similar periodic features to Wk. The difference is the peak values for the tri-axial accelerations and angular velocities. Besides, the Forward fall is quite similar to Bw-Fall, so Forward fall was not included in our activities.

Since it is very dangerous for the elderly to do simulated falls, there was no subject who was over 45 years old. Additionally, a spongy cushion was used to protect the subjects from injury during the simulated fall. While lots of injurious falls take place on hard materials (e.g., floors), the spongy cushion can absorb the impact. Hence, the acceleration values of the simulated falls could not reflect a real-world fall. Finally, in order to reduce the effect of individually-different behavior on the classification, subjects were asked to do the experiment according to the fall simulation protocol [31]. Although there were no warm up trials for subjects to familiarize themselves with the spongy cushion, the subjects knew that they would fall. This leads to the anticipation that subjects may change their postural control and response mechanisms. Just as Klenk et al. summarized, algorithms calculated on the basis of fall simulations in healthy young subjects lack the necessary accuracy requirements for real-world fall detection [30].

Because of accessibility problems for the elderly and other difficulties (e.g., cost), the amount of recorded, documented and published real-world fall data for older people is very small. Schwicker et al. made a systematic review of a total number of 96 articles on fall detection with body-worn sensors published between 1998 and 2012. It showed that less than 7% of studies have used fall data recorded from elderly people in real life, and simulated fall data were used in 90 (93.8%) studies. However, recently, the FARSEEING project, which is a collaborative European project, has set a goal of generating a large meta-database of real-world fall signals [32]. Furthermore, Vavoulas et al. introduced the smart phone to build a MobiFall dataset (an initial evaluation of fall decetion algorithms using smaetphones), which includes signals recorded from the accelerometer and gyroscope sensors for four different falls and nine different ADLs [33]. The aim of the MobiFall dataset is helpful in testing new methods and performing objective comparisons between different algorithms for fall detection and activity recognition. Nevertheless, we could not download the MobiFall dataset from the website recommended by the author. The system presented in this paper will be verified as soon as a database of real-world falls can be accessed.

## 6. Conclusions

The experiment proved that the proposed system takes advantage of wearable devices and smart phones, which are able to detect simulated falls with sufficient accuracy, and can provide timely and accurate help for the elderly. However, there is not any available data for real-world falls in China, yet. Based on the encouraging results achieved, some sensor boards, along with the software for fall detection, will be contributed to some elderly communities, so as to collect data for the daily living activities of the elderly, and harvest the database of real-world falls. Additionally, the sensor board, which integrates with Bluetooth 2.0, continuously collects and transmits the tri-axial accelerations and angular velocities with a frequency of 100 Hz. This means it requires lots of energy. For example, the sensor board with a 3.7 v, 600 mah battery can only work continuously for 4 h. Hence, the low-power technology for fall detection based on Bluetooth 4.0 will be studied in the future, which will allow the fall detection system to work for a longer time without recharging or replacing the battery. Finally, classification has been significantly improved by deep learning algorithms recently; we will research methods based on deep learning to improve the accuracy of fall detection.

## Figures and Tables

**Table 1:** AR parameters and the final FPE for tri-axial accelerations.

*a*	*x*-Axis			*y*-Axis			*z*-Axis		
	AR(1)	AR(2)	AR(3)	AR(1)	AR(2)	AR(3)	AR(1)	AR(2)	AR(3)
*a*_1_	0.9974	0.5043	0.3319	1	0.5112	0.3390	0.9953	0.5067	0.3482
*a*_2_		0.4944	0.3185		0.4888	0.3080		0.4906	0.3283
*a*_3_			0.3488			0.3526			0.3218
FPE	4.3205 × 10^−5^	3.2531 × 10^−5^	2.8591 × 10^−5^	3.2978 × 10^−5^	2.5112 × 10^−5^	2.1998 × 10^−5^	5.3838 × 10^−5^	4.0862 × 10^−5^	3.6631 × 10^−5^

**Table 2:** AR parameters and the final FPE for tri-axial angular velocities.

*ω*	*x*-Axis			*y*-Axis			*z*-Axis		
	AR(1)	AR(2)	AR(3)	AR(1)	AR(2)	AR(3)	AR(1)	AR(2)	AR(3)
*ω_1_*	1	0.6655	0.5869	0.9269	0.6346	0.5660	0.9997	0.6819	0.6251
*ω_2_*		0.3345	0.1780		0.3154	0.1767		0.3179	0.1972
*ω_3_*			0.2350			0.2182			0.1776
FPE	0.0012	0.0011	0.0010	0.0010	9.3156 × 10^−4^	8.8745 × 10^−4^	0.0011	9.7509 × 10^−4^	9.4564 × 10^−4^

**Table 3:** Experiment results with Kalman filter.

Test	Total	Correct	Wrong	Accuracy
Wk	100	100	0	100.00%
Sq	100	91	9	91.00%
Sd	100	93	7	93.00%
Bw	100	96	4	96.00%
Sd-Fall	100	95	5	95.00%
Bw-Fall	100	99	1	99.00%

**Table 4:** Experiment results without Kalman filter.

Test	Total	Correct	Wrong	Accuracy
Wk	100	100	0	100.00%
Sq	100	90	10	90.00%
Sd	100	87	13	85.00%
Bw	100	95	5	96.00%
Sd-Fall	100	94	6	94.00%
Bw-Fall	100	98	2	97.00%

**Table 5:** Accuracy comparison with different numbers of features.

The Number of Features	Accuracy	Sensitivity	Specificity	TP	FP
3	89.67%	99.50%	93.75%	0.897	0.021
7	94.50%	98.00%	93.75%	0.945	0.011
9	95.67%	99.00%	95.00%	0.957	0.009

**Table 6:** Comparing Bayes Network with other learning algorithms.

Algorithm	Accuracy	Sensitivity	Specificity	Time(s)
*k*-NN; *k* = 7	95.50%	97.00%	96.00%	<0.01
Naïve Bayes	95.50%	99.50%	94.25%	0.24
Bayes Network	95.67%	99.00%	95.00%	1.33
C4.5 Decision Tree	92.33%	99.00%	91.50%	1.7
Bagging	92.17%	99.00%	92.75%	6.11
